# ER Stress Activates the NLRP3 Inflammasome: A Novel Mechanism of Atherosclerosis

**DOI:** 10.1155/2019/3462530

**Published:** 2019-10-07

**Authors:** Xinnong Chen, Xiaochen Guo, Qihui Ge, Yixuan Zhao, Huaiyu Mu, Junping Zhang

**Affiliations:** First Teaching Hospital of Tianjin University of Traditional Chinese Medicine, Tianjin 300193, China

## Abstract

The endoplasmic reticulum (ER) is an important organelle that regulates several fundamental cellular processes, and ER dysfunction has implications for many intracellular events. The nucleotide-binding oligomerization domain-like receptor family, pyrin domain-containing 3 (NLRP3) inflammasome is an intracellularly produced macromolecular complex that can trigger pyroptosis and inflammation, and its activation is induced by a variety of signals. ER stress has been found to affect NLRP3 inflammasome activation through multiple effects including the unfolded protein response (UPR), calcium or lipid metabolism, and reactive oxygen species (ROS) generation. Intriguingly, the role of ER stress in inflammasome activation has not attracted a great deal of attention. In addition, increasing evidence highlights that both ER stress and NLRP3 inflammasome activation contribute to atherosclerosis (AS). AS is a common cardiovascular disease with complex pathogenesis, and the precise mechanisms behind its pathogenesis remain to be determined. Both ER stress and the NLRP3 inflammasome have emerged as critical individual contributors of AS, and owing to the multiple associations between these two events, we speculate that they contribute to the mechanisms of pathogenesis in AS. In this review, we aim to summarize the molecular mechanisms of ER stress, NLRP3 inflammasome activation, and the cross talk between these two pathways in AS in the hopes of providing new pharmacological targets for AS treatment.

## 1. Introduction

The endoplasmic reticulum (ER) is the primary intracellular site for protein synthesis and processing, as well as the primary calcium reservoir that maintains calcium homeostasis [1, 2]. Additionally, there are many rate-limiting enzymes located in the ER membrane involved in the synthesis of steroids and different lipids [3]. Disturbances in ER protein homeostasis lead to ER stress, which then activates the unfolded protein response (UPR). The UPR then regulates many components of the secretory pathway to restore protein homeostasis, including protein folding, maintenance of calcium homeostasis, and lipid synthesis [4, 5]. In turn, abnormal lipid and calcium metabolisms are important contributors to ER stress [6].

The nucleotide-binding oligomerization domain-like receptor family, pyrin domain-containing 3 (NLRP3) inflammasome is a type of macromolecular complex that can activate caspase-1, leading to pyroptosis. It can also induce the maturation and secretion of interleukin-1*β* (IL-1*β*) and IL-18 [7, 8]. Under pathological conditions, NLRP3 inflammasome activation is initiated by host recognition of pathogen-associated molecular patterns (PAMPs) or danger-associated molecular patterns (DAMPs) [9]. In addition, several signaling pathways, including ER stress, are also involved in the activation of the inflammasome [8].

When ER stress is excessive, calcium homeostasis, protein processing, and lipid metabolism are disrupted, which inevitably damages the intracellular microenvironment and eventually affects the activation of the NLRP3 inflammasome. In this review, we present some of the interesting cross talk in the molecular signaling pathways between ER stress and the NLRP3 inflammasome. We propose that the ER, similar to the mitochondria, is an organelle that is effective in the activation of the NLRP3 inflammasome, thus operating as a previously uncharacterized stress “rheostat” that controls pyroptosis.

Atherosclerosis (AS) is a chronic inflammatory disease that is the main pathological basis of ischemic cardiovascular and cerebrovascular diseases [10–12]. Several studies have documented that both the NLRP3 inflammasome and ER stress closely affect the progression of AS [13, 14]. Since there are multiple links between ER stress and the NLRP3 inflammasome, it is not inconceivable that these links may also be related to AS. Recognition of the potential direct or indirect links between these divergent pathogenic processes may offer new avenues for the development of treatments against AS.

## 2. Control of ER Homeostasis: Mechanisms and Function

### 2.1. Protein Synthesis, Folding, and Degradation

The ER serves as a platform that mediates the synthesis and folding of 30% of the proteome, but its normal function is easily influenced by external factors [15–17]. Because of the complex and crucial task of protein synthesis and modification, a protein quality control mechanism in the ER is required to ensure protein homeostasis in cells. In fact, at least a third of all polypeptides translocated into the ER fail to satisfy the quality control mechanisms. These cargoes that do not reach their final destination are degraded via the ER-associated degradation (ERAD) pathway which removes misfolded/unfolded proteins to the cytosol for subsequent ubiquitination and degradation by the proteasome [16–18]. If ubiquitination and proteasomal degradation are impaired, then misfolded/unfolded proteins will continue to accumulate in the ER and eventually clog the ER lumen [17, 19]. Under stress conditions, the demand for secreted and membrane proteins rapidly increases, resulting in increased levels of protein synthesis that exceed the protein degradation capacity of cells, which then leads to protein accumulation [15, 19–22]. Insults caused by genetic, environmental, or nutritional factors induce imbalances in the ER quality control mechanism, leading to the accumulation of proteins in nonnative conformations [15, 19, 21–23]. The situation becomes even more critical if dysregulations in the oxidation-reduction balance, calcium levels, or posttranslational modifications are present [15, 17, 19, 21, 22]. In addition, deficiencies in autophagy, energy deprivation, and inflammatory stimulation all lead to the accumulation of misfolded proteins [19]. To summarize, due to high protein load on the organelles or impaired ER quality control mechanisms, protein degradation can become blocked, leading to protein accumulation in the ER, which induces the UPR and thereby initiates a stress response that restores cellular homeostasis [15, 16].

### 2.2. UPR Signal Transduction

**Figure 1:**
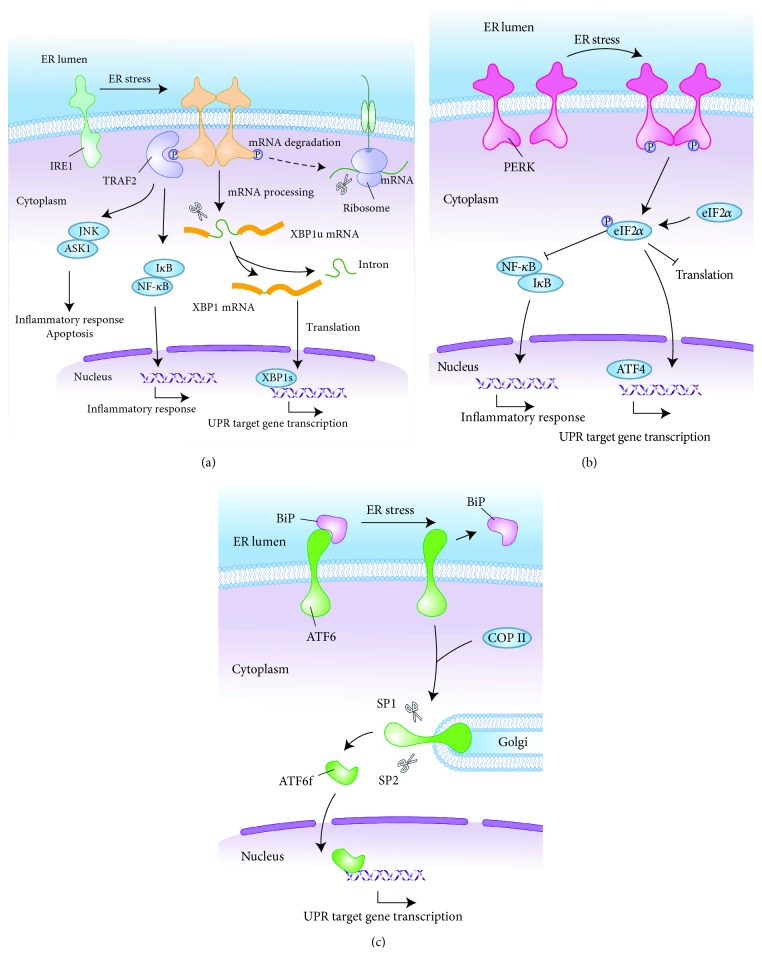
UPR signaling pathways. UPR induced by ER stress triggers downstream signaling through three major sensing proteins (IRE1, PERK, and ATF6). (a) IRE1 autophosphorylation induces XBP1-specific cleavage, enhancing ER folding function and UPR gene transcription. Furthermore, activated IRE1 recruits TRAF2 which induces apoptosis and inflammation through JNK and NF-*κ*B pathways. IRE1*α* also degrades select mRNAs through RIDD. (b) Activated PERK phosphorylates eIF2*α* which upregulates ATF4 expression to promote UPR gene transcription while inducing NF-*κ*B-mediated inflammatory responses. (c) ATF6 interacts with COPII to transport ATF6 to the Golgi for cleavage, and the resulting ATF6f induces the transcription of downstream genes such as XBP1 and CHOP. UPR: unfolded protein response; ER: endoplasmic reticulum; IRE1: inositol-requiring enzyme 1; PERK: protein kinase RNA- (PKR-) like kinase; ATF6: activating transcription factor 6; XBP1: X-box-binding protein-1; TRAF2: tumour necrosis factor receptor- (TNFR-) associated factor-2; JNK: Jun-N-terminal kinase; NF-*κ*B: nuclear factor *κ*B; RIDD: regulated IRE1-dependent decay; eIF2*α*: eukaryotic translation initiation factor 2*α*; ATF4: activating transcription factor 4; COPII: coat protein II; CHOP: CCAAT/enhancer-binding protein-homologous protein.

As the most important response in the ER stress transduction pathway, the UPR has been studied in depth over the past decade. Three highly conserved proximal effectors of UPR, namely, inositol-requiring enzyme 1 (IRE1), protein kinase RNA- (PKR-) like kinase (PERK), and activating transcription factor 6 (ATF6), coordinate the cell-autonomous response to ER stress [4]. In the absence of stress, these ER-localized transmembrane proteins are coupled to the ER chaperone immunoglobulin-binding protein (BiP) and remain in an inactive state. During ER stress, BiP separates from stress signal transducers and preferentially chaperones unfolded/misfolded proteins, thereby permitting IRE1, PERK, and ATF6 to convert to their active states [24]. The UPR is then triggered by these activated protein sensors and their downstream transcriptional effectors via three distinct pathways (see Figure 1).

In response to ER stress, IRE1 is activated by transautophosphorylation at its cytosolic kinase domain, eliciting endoribonuclease activity that mediates sequence-specific cleavage of the mRNA encoding X-box-binding protein-1 (XBP1). After endoribonuclease cleavage, unspliced XBP1 (XBP1u) converts to spliced XBP1 (XBP1s) which is a potent transcriptional activator that augments the protein folding capacity of ER [25]. In addition, IRE1 induces the transcription of UPR genes that promote ERAD via XBP1 mRNA splicing to restore homeostasis and cytoprotection [26]. Activated IRE1 kinase interacts with tumour necrosis factor receptor- (TNFR-) associated factor-2 (TRAF2), which leads to the activation of apoptotic signaling kinase-1 (ASK-1) and the downstream factor Jun-N-terminal kinase (JNK); the latter of which is a member of the mitogen-activated protein kinase (MAPK) family that regulates inflammation and apoptosis [27, 28]. In addition to ASK-1 and JNK, the activation of IRE1*α* can also contribute to cell death through sustained regulated IRE1-dependent decay (RIDD), which is a process in which IRE1*α* RNase activity degrades a subset of mRNAs [4, 29]. IRE1-TRAF2 complexes also recruit I*κ*B kinase (IKK), resulting in the phosphorylation and degradation of I*κ*B, as well as consequent translocation of nuclear factor-*κ*B (NF-*κ*B) into the nucleus to regulate the transcription of inflammatory genes [30].

Similarly, PERK dissociated from BiP is also responsible for decreasing ER workload by inhibiting mRNA translation, thereby further decreasing protein synthesis. Activated PERK phosphorylates eukaryotic translation initiation factor 2*α* (eIF2*α*), which greatly inhibits general translation by interfering with 5′cap assembly, facilitating the accumulation of ATF4 through an alternative translation initiation site [31]. ATF4 transcriptionally upregulates CCAAT/enhancer-binding protein-homologous protein (CHOP) and growth arrest and DNA damage-inducible 34 (GADD34) which participates in a feedback loop to dephosphorylate eIF2*α* [32, 33]. In addition, PERK-eIF2-mediated translational suppression of I*κ*B increases the activity of NF-*κ*B which subsequently transcribes a broad network of proinflammatory signals [34, 35].

After separating from BiP, ATF6 interacts with the coat protein II (COPII) complex, following which ATF6 is transited to the Golgi apparatus where it is consecutively cleaved by site 1 protease (S1P) and S2P. As a result, ATF6f, which is a cytosolic domain fragment of ATF6, is liberated from the membrane and translocated into the nucleus [36, 37]. ATF6f contains a basic leucine zipper domain which acts as a transcription factor to regulate transcription activation of specific target genes involved in protein folding and ERAD, such as CHOP and XBP1 [25, 38]. As ATF6 is capable of activating inflammation-related proteins such as C-reactive protein and NF-*κ*B, it would be interesting to assess ATF6 as a synergistic mediator between the ER stress and proinflammatory signaling pathways [39].

### 2.3. Secretory Pathways

The ER and UPR are crucial in the maintenance of basic functions of many cells; in addition to their well-known role in protein quality control, they are highly important for many aspects of the secretory pathway in restoring protein folding homeostasis, including maintenance of calcium homeostasis, ROS production, and lipid synthesis [4, 40]. Calcium stored in the ER plays a key role in posttranslational processing, folding, and export of proteins, as well as in Ca^2+^ signaling [41]. The accumulation of misfolded proteins in the ER can interfere with Ca^2+^ homeostasis, and conversely, a change in Ca^2+^ content in the lumen has a major effect on protein synthesis [42]. Some new proteins found at the ER-mitochondria interface have drawn attention as pivotal targets for regulating interorganelle calcium signaling potentially leading to mitochondrial Ca^2+^ overload and apoptotic cell death [43–46]. Among this group, it is not fully understood which are specifically involved during ER stress. The study identified that a sarcoendoplasmic reticulum Ca^2+^-ATPase 1 (SERCA1) variant (S1T) acting as an ER stress protein was directly involved in Ca^2+^-dependent mitochondrial apoptosis. In addition, S1T was found to amplify ER stress through the PERK-eIF2*α*-ATF4-CHOP pathway [41]. ER stress-inducible eIF2*α* kinase PERK is also involved in the activation of the integrated stress response (ISR), which is important in dealing with physiological levels of ER stress [47]. ROS has a dual role in ER stress signaling that can be loosely described as the signaling intermediates that report ER stress to the UPR in order to mitigate ER stress but appear to arise and contribute to cell death in chronic ER stress [48].

The ER is the central hub of lipid metabolism, as most of lipogenesis occurs on the cytoplasmic surface of the ER membrane, including the synthesis of triacylglycerols, sterols, ceramides, and phospholipids, as well as that of lipid droplet biogenesis [5, 49]. Additionally, the ER is the site of fatty acid desaturation [5]. Recent studies show that the UPR can directly control the transcription of genes coding for proteins involved in lipid metabolism and interfere with the secretion of apolipoproteins [50, 51]. UPR stress sensors can be activated by lipotoxic stress in addition to classical protein folding stress [52, 53]. A recent study indicates that certain stress stimuli which cause lipid- or membrane-related aberrations are likely to be sensed by IRE1, without the need for interaction between IRE1 and unfolded proteins [54]. Furthermore, membrane lipid saturation induces autophosphorylation of IRE1*α* and PERK, which is different from the mechanism by which unfolded proteins activate the UPR [55–57]. A previous study has demonstrated that ER stress can dysregulate lipid metabolism, leading to lipid disorders by activating the sterol regulatory element-binding proteins (SREBPs) [58]. Both SREBP-1 and the homologous SREBP-2 are inserted into the ER/nuclear membrane [59]. Within the ER membrane, SREBP cleavage-activating protein (SCAP) interacts with the newly synthesized SREBP precursor and insulin-induced gene (Insig). SREBP-1 and SREBP-2 contribute to cholesterol and fatty acid homeostasis through transcriptional regulation of genes involved in the biosynthesis of cholesterol, triacylglycerides, and phospholipids [60]. Inhibition of SREBP-1 prevents excessive lipid accumulation via downregulation of the expression of its downstream proteins [61]. SREBP-2 is a major regulator of cholesterol biosynthesis [60]. When cholesterol is depleted, the expression of SREBP-2 along with that of miR-33, which is located at an SREBP-2 intron, increases to replenish cellular cholesterol [62]. In addition, interactions among sterol metabolism, ISR, and the SREBP pathway affect lipid metabolism as well [10, 47]. In summary, these results suggest that lipids, calcium, and ROS, as products of secretion pathways, can be activated by different ER stress signals to mediate the information transmission between the ER and other organelles, but the specific mechanisms are far from being spelled out.

## 3. Molecular Mechanisms of NLRP3 Inflammasome Activation

The NLRP3 inflammasome is a cytosolic protein complex composed of the sensor protein NLRP3, the adaptor protein known as apoptosis-associated speck-like protein containing a C-terminal caspase recruitment domain (ASC), and the effector molecule caspase-1 [63, 64]. NLRP3 recruits ASC upon activation, which serves as a platform for the recruitment and autocatalytic cleavage of pro-caspase-1, giving rise to active caspase-1 [65, 66]. Once activated, caspase-1 promotes IL-1*β* and IL-18 maturation and release and also cleaves gasdermin D (GSDMD). The N-terminal domain of GSDMD then becomes bound to the plasma membrane inner leaflet, forming many pores on the host cell membrane, which directly destroys membrane permeability, leading to pyroptosis and passive release of proinflammatory cytokines, such as mature IL-1*β* and IL-18 [7, 8].

It is generally believed that NLRP3 activators do not directly interact with NLRP3 but induce one or more downstream cellular activities or disorders [8]. The activation of the NLRP3 inflammasome requires two signals: toll-like receptor 4 (TLR4) ligand lipopolysaccharide (LPS) binding to its receptor, which induces the transcriptional upregulation of NLRP3 along with pro-IL-1*β* through NF-*κ*B (signal 1). Alternatively, TLR4 provides signal 1 by means of its adaptors myeloid differentiation factor 88 (MyD88), interleukin 1 receptor-associated kinase 1 (IRAK1), and IRAK4, independently of new protein synthesis [67]. A posttranscriptional modification, such as NLRP3 deubiquitination mediated by BRCA1/BRCA2-containing complex subunit 3 (BRCC3), is required for NLRP3 activation (signal 2) [67, 68]. The second signal provided by NLRP3-activating agents (e.g., ATP, ROS, oxidized mitochondrial DNA (mtDNA), and other stimuli) triggers assembly and activation of the NLRP3 inflammasome, followed by proinflammatory caspase-mediated pyroptosis [69–71].

## 4. The Molecular Pathways between ER Stress and NLRP3 Inflammasome Activation: Mechanistic Cross Talk with AS

A great deal of research indicates that ER stress occurs in a variety of cell types involved in AS including endothelial cells and macrophages and also influences the disease process of AS by coordinating protein and lipid metabolism, inflammatory response, various stress responses, and cell death [13, 72–78]. Similarly, the NLRP3 inflammasome and its genetic variants are involved in atherosclerotic pathogenesis [79, 80]. The mechanism of pathogenesis involves mediating immune cell interactions, driving sterile inflammation, and promoting the progression of atherosclerotic plaques, such as that seen in AS [14, 79, 81, 82]. Moreover, the NLRP3 inflammasome can instigate inflammatory pathologies toward hyperhomocysteinemia-aggravated AS [83, 84]. Therapeutic approaches targeting ER stress and the NLRP3 inflammasome separately have shown promise in the prevention and/or regression of AS. There are multiple associations between ER stress and the NLRP3 inflammasome, and a variety of cellular processes observed among these associations are required for atherogenesis, which sheds lights on the significance of AS therapies targeting these associations.

### 4.1. Terminal Signaling in the UPR

#### 4.1.1. p38 MAPK

Previous studies have shown that the UPR induces an increase in p38 MAPK activation [85, 86]. In particular, ER stressors lead to PERK-dependent activation and recruitment of MAPK kinase 4 (MKK4) to lysosomes, activating p38 MAPK at the lysosomes [87]. Under the action of ASK1, IRE1 can also activate p38 MAPK [88]. In addition, p38 MAPK modulates the UPR via p38-dependent phosphorylation of CHOP and ATF6 [86]. So, p38 MAPK plays a dual role in the UPR [89]. The p38 MAPK pathway participates in maintaining a normal cell cycle, differentiation, apoptosis, and expression of inflammatory cytokines and chemokines. Studies have confirmed that high mobility group box-1 (HMGB1) promotes the synthesis of pro-IL-1 and pro-IL-18 by activating p38 MAPK [90]. ASK1 can increase the apoptosis of macrophages and inhibit AS induced by hyperlipidemia, but the plaque vulnerability is significantly increased by augmenting the area of plaque necrosis [91]. The p38*α* MAPK is the most widely expressed subtype in the p38 MAPK family that is closely related to the occurrence and development of AS. Selective inhibition of p38*α* MAPK produces multifaceted effects on foam cell formation, apoptosis, and cytokine induction and prevents the inflammatory cascade in AS [92].

#### 4.1.2. JNK

During severe ER stress conditions, sustained IRE1*α* oligomerization can recruit the adaptor protein TRAF2, which serves as an activation platform for ASK1 and its downstream target JNK. Dominant-negative TRAF2 inhibits the activation of JNK by IRE1 [28, 93]. E3 ligase carboxyl terminus of HSC70-interacting protein- (CHIP-) regulated IRE1*α* ubiquitination increases JNK signaling without affecting XBP1 mRNA splicing [27]. As an IRE1-interacting/modulator protein, N-Myc interactor (NMI) negatively modulates IRE1-dependent activation of JNK and apoptosis [94]. Additional experiments implicate the PERK/eIF-2*α* signaling pathway as a contributor to JNK activation [95]. JNK input is not limited to upstream of ER stress, but also downstream, as the abrogation of JNK attenuates ER stress [96, 97]. Hara et al. found that JNK is required for the activation of caspase-1 via the NLRP3 inflammasome. Inhibition of JNK abolishes the formation of ASC specks without affecting the interaction of ASC with NLRP3, which suggests that JNK acts upstream of ASC phosphorylation [98]. JNK inhibitors decrease the activation of caspase-1 and reduce circulating amounts of IL-1*β*. Moreover, JNK can phosphorylate B-cell lymphoma-2 (Bcl-2) family proteins (such as Bcl-2 and Bcl-XL) to regulate NLRP3 inflammasome activation [99]. Several studies have identified that JNK2 knockout leads to decreased incidence of AS *in vivo* compared to JNK1 knockout. Macrophages lacking JNK2 inhibit the phosphorylation of scavenger receptor A (SR-A) and foam cell formation [100]. However, the absence of JNK1 in macrophages can prevent apoptosis and increase cell survival, which promotes the formation of early AS [101].

#### 4.1.3. XBP1

XBP1 is the downstream effector molecule of IRE1 and ATF6 [25]. XBP1 can control the activation of the NLRP3 inflammasome. For example, Robblee et al. proved that XBP1 plays a mediating role in the process of IRE1 regulation of saturated fatty acid (SFA) metabolism to activate the NLRP3 inflammasome in macrophages. Interference of XBP1 gene coding or transcription is a new method by which the activation of the NLRP3 inflammasome may be controlled [102]. XBP1 can inhibit NLRP3 activity and caspase-1 and IL-1*β* release, as well as mRNA synthesis [102–104]. Multiple studies have found that XBP1 is involved in the development of AS, and excess amounts of XBP1 expression can be observed in the arterial branch points and plaques of ApoE^−/−^ mice. XBP1 regulates macrophage death, foam cell formation, and IL-8 and TNF*α* release, as well as inducing endothelial cell apoptosis, autophagy, proliferation, and smooth muscle cell calcification. In addition, XBP1 interferes with lipid metabolism and XBP1 deletion significantly reduces plasma cholesterol levels in ApoE^−/−^ mice. In conclusion, the continuous activation of XBP1 promotes the formation of AS [105–107].

#### 4.1.4. CHOP

When the UPR fails to alleviate ER stress, apoptosis occurs mainly via CHOP [108]. CHOP is a transcription factor that promotes apoptosis. When the UPR is activated, PERK promotes CHOP expression by increasing the content of the downstream signaling protein ATF4 [32]. ATF6 can also directly regulate CHOP [109]. The IRE1-XBP1 signaling pathway increases CHOP expression by activating JNK [110]. In addition to inducing apoptosis, CHOP overexpression can also activate the NLRP3 inflammasome, leading to pyroptosis, as well as the secretion of IL-1*β*, caspase-1, and caspase-11 [108]. Many researchers have utilised CHOP to investigate the relationship between apoptosis and AS. What is more, previous experiments have confirmed that the ER stress effector CHOP is related to plaque necrosis [111]. CHOP expressed in vascular cells contributes to the progression of vascular remodeling and AS [112].

#### 4.1.5. NF-*κ*B

Branches of characteristic sensor pathways (IRE1, PERK, and ATF6) involved in the UPR have been reported to regulate the NF-*κ*B pathway [113]. ER stress primes cells to promote the secretion of IL-1*β* by activating NF-*κ*B to express pro-IL-1*β* [114]. The ER stress inhibitor 4-phenylbutyric acid (4-PBA) reduces the release of proinflammatory factors such as IL-1*β* by inhibiting the NF-*κ*B signaling pathway [115]. NF-*κ*B upregulates IL-1*β* and NLRP3 in a TLR-independent pathway [116]. In addition, studies have confirmed that NF-*κ*B upregulates expression of cyclooxygenase 2 (COX-2) which can activate the NLRP3 inflammasome to induce IL-1*β* secretion and pyroptosis [117, 118]. NF-*κ*B expression is increased in many inflammatory diseases, and its activation can be used for both protective and destructive outcomes. A study has shown that NF-*κ*B plays an important regulatory role in AS and NLRP3 can affect NF-*κ*B and its downstream signaling pathway, leading to the occurrence of AS [71]. NF-*κ*B can induce endothelial dysfunction by stimulating the release of some inflammatory mediators, including IL-6 and TNF-*α* [119]. Moreover, as the downstream gene of NF-*κ*B, COX-2 plays a role in promoting AS. Inhibiting COX-2 expression significantly reduces early atherosclerotic lesion formation [120].

#### 4.1.6. Thioredoxin-Interacting Protein (TXNIP)

TXNIP is an important junction that links oxidative stress to inflammation. In response to ROS, TXNIP dissociates from thioredoxin (TRX) and binds to NLRP3, leading to the activation of the NLRP3 inflammasome which results in the maturation and release of IL-1*β* and IL-18 [121]. TRX80, a C-terminally truncated form of TRX-1, can also activate the NLRP3 inflammasome and release potent atherogenic cytokines IL-1*β* and IL-18 [122]. Previous studies have shown that TXNIP is closely related to the activation of the inflammasome under oxidative stress, but it has recently been found that TXNIP is an important molecular node linking ER stress to inflammation.

TXNIP can be induced by the IRE1 and PERK-eIF2*α* pathway to induce transcription of IL-1*β* mRNA. In addition, it also activates the NLRP3 inflammasome to release IL-1*β* and regulate ER stress-related cell death [123]. ER stress-induced ROS activates the NLRP3 inflammasome through TXNIP, leading to IL-1*β* secretion [114]. A study has suggested that ER stress has an effect on inflammasome activation and that TXNIP plays an important role in ER stress-mediated promotion of IL-1*β* maturation [123]. Lerner et al. found that TXNIP is a significant node of terminative UPR. Hyperactivity of IRE1*α* increases the stability of TXNIP mRNA by reducing the level of microRNA-17 (miR-17), which normally leads to translational suppression of TXNIP, which in turn increases the expression of TXNIP protein, thereby activating the NLRP3 inflammasome, leading to the dissociation of caspase-1 and the secretion of IL-1*β* [124]. It was found that caspase-2 activation takes place via TXNIP, which results in mitochondrial dysfunction and cytochrome C release. After mitochondrial injury, DAMP is released to activate the inflammasome and to produce IL-1*β*. Furthermore, caspase-2 is able to activate caspase-1 [125, 126].

Byon et al. showed that atherosclerotic plaques in the aortic root decrease by 49% and abdominal aortic lesions decrease by 71% in TXNIP-ApoE-double-knockout mice, compared to control ApoE-knockout mice [127]. The data show that TXNIP plays a key role in the oxidization, inflammation, and the development of AS in mice. Intervention against TXNIP expression may be a potential target for the prevention and treatment of AS and of inflammatory vascular disease. In addition to oxidative injury and inflammation, TXNIP can increase intimal thickness in the carotid artery and lead to abnormal glucose metabolism. A study among the Chinese Han population reported that TXNIP single-nucleotide polymorphisms independently and gradually increase the risk of coronary heart disease by regulating TXNIP expression and gene-environment interactions [128].

#### 4.1.7. The Mammalian/Mechanistic Target of Rapamycin Complex 1 (mTORC1)

The mTOR protein is a master manipulator of cell growth and metabolism. This kinase contains two protein complexes, mTORC1 and mTORC2, which execute distinct cellular responses. Multiple studies have found that uncontrolled mTORC1 signaling is intertwined with ER stress [129, 130]. Uncontrolled mTORC1 signaling is known to promote dysregulated ER stress-UPR [129, 131, 132] and may mediate ER stress and lipogenesis by regulating SREBP signaling [54, 133, 134]. Besides, ER stress also plays a role in regulating mTORC1. A study found that ATF6 induces Ras homologue enriched in brain (RHEB) which is an activator of mTORC1, thus activating mTORC1 [135]. The PERK-ATF4 pathway induces the expression of regulated in development and DNA damage 1 (REDD1) and tribbles homolog 3 (TRB3), both of which lead to mTORC1 suppression [131, 136–139]. In addition, the PERK signaling pathway can induce sestrin-2, thus inhibiting mTORC1 to maintain ER homeostasis [140, 141].

Moon et al. demonstrated that mTORC1-induced hexokinase 1- (HK1-) dependent glycolysis regulates NLRP3 inflammasome activation in macrophages, suggesting that mTORC1 is a potent NLRP3 inflammasome inducer [142]. Additionally, mTOR activates the inflammasome partially via ROS-induced NLRP3 expression [143]. The mTORC1 inhibitor REDD1 regulates the priming of the NLRP3 inflammasome through a NF-*κ*B-dependent pathway [144].

Several mechanisms of mTORC1 inhibition are involved in the early stages of atherogenesis [145]. First, mTORC1 activity contributes to SREBP-2-mediated cholesterol uptake, which facilitates AS progression [146]. SREBP-2 is involved in regulating cholesterol metabolism in macrophages, creating an immunometabolic circuit that links perturbations in cholesterol biosynthesis with innate immune activation [147], while mTORC1 may promote lipid uptake and foam cell formation [148, 149]. Second, mTOR silencing induces macrophage autophagy, which is a potential strategy for the treatment of atherosclerotic plaques [150]. Third, the inhibition of mTORC1 leads to the release of large amounts of cytokines and the shift of macrophages to a hyperinflammatory state [151–153]. However, in contrast to *in vitro* findings, mTORC1 inhibition decreases monocyte migration and reduces proinflammatory cytokines in plasma, both of which are involved in plaque development [145, 154]. In addition, mTORC1 may also participate in the AS process by regulating vascular smooth muscle cell proliferation, endothelial dysfunction, and neoangiogenesis [155–157].

### 4.2. Mediating Effect of ROS

**Figure 2:**
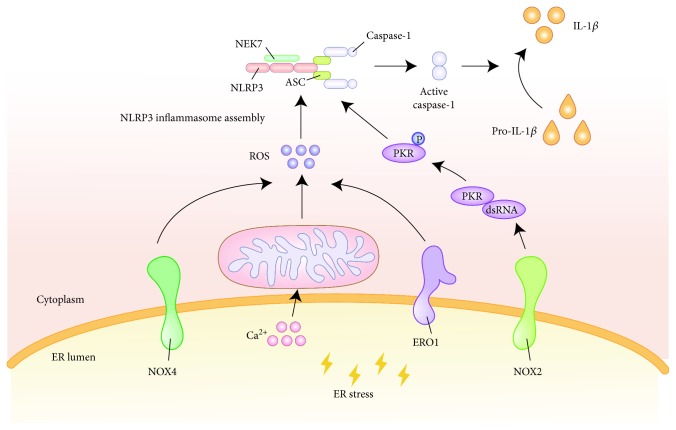
The effect of ROS in the process of ER stress activation of the NLRP3 inflammasome. ER induces ROS production via NOX4 and ERO1 during stress. The release of Ca^2+^ in the ER causes mitochondrial damage which further aggravates the release of ROS. The increased ROS level triggers NLRP3 inflammasome assembly. In addition, NOX2 regulates dsRNA-activated PKR expression under ER stress and affects the process of NLRP3 inflammasome activation. ROS: reactive oxygen species; ER: endoplasmic reticulum; NLRP3: nucleotide-binding oligomerization domain-like receptor family, pyrin domain-containing 3; NOX4: NADPH oxidase 4; ERO1: ER oxidase 1; NOX2: NADPH oxidase 2; PKR: protein kinase RNA.

ROS play a significant role in oxidative stress, inflammation, apoptosis, cell growth, alteration in vascular tone, and oxidation of low-density lipoprotein cholesterol (LDL-C) [158, 159]. Previously, ROS have been considered to be a type of marker of oxidative stress, but more recently, researchers have found that ROS play a dual role in ER stress signaling. During ER stress, NADPH oxidase (NOX) located in the ER can induce ROS production in order to coordinate the UPR and to restore ER homeostasis [48]. NOX is composed of seven subtypes (NOX1-5 and dioxygenase 1-2) and is a type of cellular enzyme that specializes in the production of ROS [160]. IRE1 phosphorylates JNK, which partially triggers the activation of the downstream activator protein 1 (AP1), while the IRE1-JNK-AP1 signaling pathway facilitates NOX4 expression. Small interfering RNA (siRNA) silencing of IRE1 or inhibition of JNK activity can reduce their gene expression. Another study found that JNK may be a catalyst for NOX2 gene transcription [161, 162]. In the event that ER stress is not relieved over time, ER oxidase 1 (ERO1) partly induces an ROS increase [163]. Excessive ROS production in ER will cause calcium deposition in the mitochondria and further aggravate mitochondrial damage [164]. In addition, calcium transfer across ER-mitochondria protein tether sites appears to further contribute to the release of ROS [48] (see Figure 2).

ROS stimulation under oxidative stress and ER stress is essential for the activation of the NLRP3 inflammasome in macrophages, where NOX and mitochondrial ROS (mtROS) may exert an impact on the inflammasome activation [165, 166]. ROS can control the assembly and activation of the NLRP3 inflammasome as well as the secretion of IL-1*β*, which ultimately induces endothelial cell pyroptosis [167]. NIMA-related kinase 7 (NEK7) acts as an upstream ROS sensor for the detection of increasing ROS level and for triggering inflammasome assembly [168, 169]. SREBP-2 induces NOX2 transcription and NLRP3 expression, leading to IL-1*β* expression and endothelial inflammatory response [62]. NOX4 not only activates NF-*κ*B through ROS but also activates MAPK to induce the secretion of proinflammatory factors [160]. In addition, NOX2 regulates the expression of dsRNA-activated protein kinase R (PKR) under ER stress [162]. In a cell-free system, PKR autophosphorylation leads to the de novo association of NLRP3, ASC, and pro-caspase-1, resulting in inflammasome activity. PKR deficiency significantly inhibits the secretion of IL-1*β*, IL-18, and HMGB1 [170].

Among the seven subtypes of NOX, NOX1, NOX2, NOX4, and NOX5 are expressed in the vascular system. NOX1 and NOX2 can induce atherogenesis by promoting both intrinsic and extrinsic vessel wall cellular inflammation [171]. Notably, in multiple atherosclerotic mouse models, the deletion of NOX4 accelerates atherogenesis, emphasizing the diverse signaling roles served by NOX [172]. NOX4 is widely expressed in vascular smooth muscle cells and is critical for maintaining vascular homeostasis. The overexpression of this gene leads to the increase in the ROS level, senescence, and susceptibility to apoptosis which are closely related to the severity of AS [171]. NOX4 directs homeostatic UPR responses and subsequent autophagic activity, as well as preserving vascular endothelium function in response to proatherogenic ER stress, which serves primarily atheroprotective effects [48].

### 4.3. ER Stress Induced the Ca^2+^ Signaling Pathway

**Figure 3:**
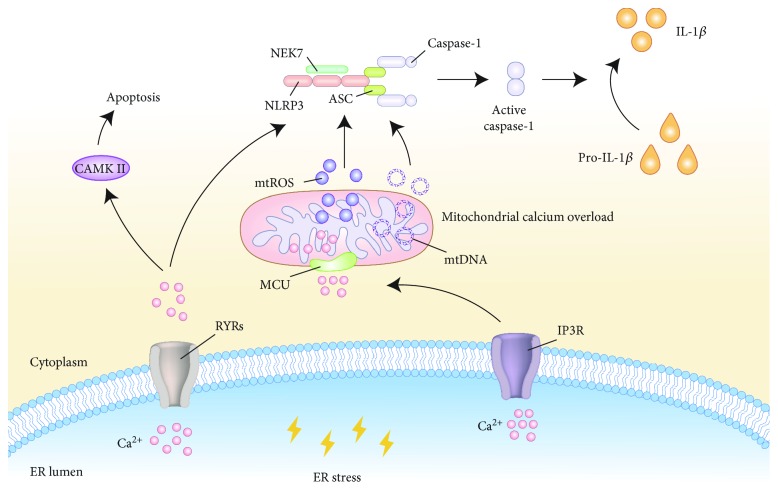
Ca^2+^ signal transduction events linking ER stress to NLRP3 inflammasome activation. During ER stress, calcium homeostasis in the ER is imbalanced and Ca^2+^ is released into the cytoplasm through two channel proteins, namely, RYRs and IP3R. Accumulated Ca^2+^ moves into the mitochondrial matrix via the MCU, which leads to mitochondrial calcium overload and organelle damage. Mitochondrial damage causes mtROS production, mtDNA release, and cardiolipin externalization, which activate the NLRP3 inflammasome. In addition, an increased Ca^2+^ level directly affects the process of inflammasome activation. ER: endoplasmic reticulum; NLRP3: nucleotide-binding oligomerization domain-like receptor family, pyrin domain-containing 3; RYRs: ryanodine receptors; IP3R: inositol 1,4,5-trisphosphate receptors; MCU: mitochondrial calcium uniporter; mtROS: mitochondrial reactive oxygen species; mtDNA: mitochondrial DNA.

As a ubiquitous second messenger of signal transduction, calcium drives complicated molecular pathways including gene expression, protein biosynthesis and secretion, cell metabolism, and apoptosis [173, 174]. The ER is the major calcium storage organelle, and ER dysfunction induces the release of calcium from the ER, which ultimately leads to cellular dysfunction. For example, a high cytosolic level of calcium activates CAMKII which then induces apoptosis through Fas signaling [175]. Ca^2+^ is released from the ER via several channels, in particular ryanodine receptors (RYRs) and inositol 1,4,5-trisphosphate receptors (IP3R) [176]. These channels tend to facilitate accumulated Ca^2+^ moving into the mitochondrial matrix via the mitochondrial calcium uniporter (MCU), leading to mitochondrial dysfunction, apoptosis, inflammasome activation, and IL-1*β* secretion [177] (see Figure 3). The ER and mitochondria are closely related in physiology and function, and they can affect the metabolism of mitochondria jointly.

Mitochondria are far more than passive Ca^2+^ sinks. Special Ca^2+^ transport mechanisms, such as the MCU, have been found to coordinate the balance between Ca^2+^ influx and efflux across the mitochondrial inner membrane in order to establish Ca^2+^ homeostasis within the cell [177]. Several findings clearly indicate that excessive ER-released Ca^2+^ results in mitochondrial calcium overload and mitochondrial injury, leading to mtROS production, cardiolipin externalization, and mtDNA release, leading to the further activation of the inflammasome and the production of IL-1*β* [178–182]. How does cytoplasmic Ca^2+^ find the balance between causing mitochondrial stress and NLRP3 activation is a question worth considering. It is possible that intracellular Ca^2+^ concentration does not reach the threshold for mitochondrial damage and NLRP3 activation under many conditions. In addition to damaging the mitochondria, Ca^2+^ mobilization directly regulates the activation of the NLRP3 inflammasome. Studies have proven that Ca^2+^ can promote spontaneous NLRP3-ASC association in cell-free lysates from LPS-stimulated macrophages [183]. In conclusion, ER-released Ca^2+^ may be a kind of common trigger for the activation of the NLRP3 inflammasome.

Previous research has established that intracellular Ca^2+^ is involved in several atherogenesis-associated processes, including abnormal contraction, differentiation and proliferation of vascular smooth muscle cells, oversecretion of extracellular matrix proteins, excessive production of chemoattractants and growth factors, platelet aggregation, and foam cell formation, as well as vascular inflammation [184, 185]. Calcium mineralization in the atherosclerotic artery lumen promotes and solidifies plaque formation, leading to vascular stenosis [174]. However, coronary calcification is associated with plaque burden but not luminal stenosis [186]. The multiethnic study of atherosclerosis (MESA) suggested that coronary artery calcium is associated closely in a graded fashion with 10-year risk of atherosclerotic cardiovascular disease (ASCVD) incident [187]. In addition, the increasing number of coronary arteries with calcified plaques indicates an enhancive incidence of “diffuse” multivessel subclinical AS [188].

### 4.4. Sterol Metabolic Pathway

The ER hosts metabolic pathways that regulate cholesterol synthesis and is the location in which cholesterol can be reesterified, allowing for cytoplasmic storage in the form of lipid droplets [3, 189]. During ER stress, the SREBP pathway is activated to maintain lipid homeostasis. As an important element of ER stress, BiP overexpression strongly reduces the expression of SREBP-2 and target genes, leading to greatly decreased hepatic cholesterol concentration. A study has shown that the suppression of insulin-induced SREBP cleavage is caused by overexpression of BiP and that the SREBP-1c complex is able to bind BiP [190]. IRE1-dependent activation of XBP1 contributes to both ER gene expression and lipid biosynthesis [191, 192]. As a potent transcription factor, XBP1s can directly transcribe lipid metabolism-related targets [193]. Ning et al. found that there is a direct interaction between XBP1 and the SREBP-1 promoter. Overexpression of the activated XBP1 increases the promoter activity of SREBP-1, while knockdown of either IRE1*α* or XBP1 prevents the insulin-stimulated promoter activity [194]. ER stress-regulated kinase, PERK, serves as an important regulator of lipid metabolism via regulation of SREBP processing [195]. PERK deletion perturbs SREBP1c Golgi processing, thereby reducing the expression of key lipogenic enzymes. PERK activation is sufficient for the activation of lipogenesis in the liver, and there is an active role for the PERK-eIF2*α* signaling pathway in the regulation of hepatic lipogenesis [51]. Furthermore, S1P and S2P enzymes that cleave ATF6 can also process SREBPs in response to cholesterol deprivation [196]. In summary, these findings indicate that the UPR is an important regulator of the SREBP pathway.

**Figure 4:**
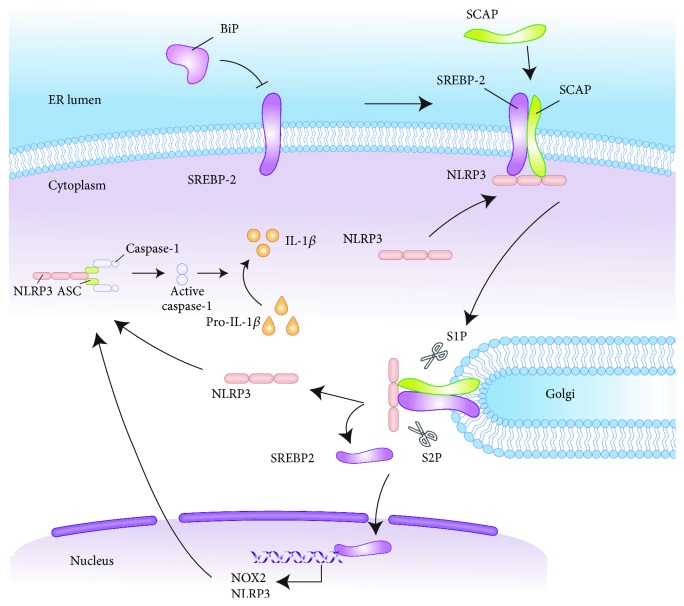
SREBP pathway mediated NLRP3 inflammasome activation. ER stress activates the SREBP pathway to maintain lipid homeostasis. NLRP3 associates with SCAP-SREBP-2 to form a ternary complex which translocates from the ER to the Golgi, where the complex is cleaved by S1P and S2P. The cleaved NLRP3 can be used for inflammasome assembly. SREBP-2, on the other hand, stimulates NOX2 and NLRP3 expression transcriptionally which in turn affects the inflammasome activation process. As an important ER stress factor, BiP overexpression affects ER lipid metabolism via significantly inhibiting SREBP-2 and downstream target gene expression. SREBP: sterol regulatory element-binding protein; NLRP3: nucleotide-binding oligomerization domain-like receptor family, pyrin domain-containing 3; ER: endoplasmic reticulum; SCAP: SREBP cleavage-activating protein; S1P: site 1 protease; S2P: site 2 protease; NOX2: NADPH oxidase 2; BiP: binding protein.

In addition to controlling cholesterol biosynthesis, the SCAP-SREBP-2 complex serves as a signaling hub integrating cholesterol metabolism with NLRP3 inflammasome activation (see Figure 4). Mechanistically, NLRP3 associates with SCAP-SREBP-2 to form a ternary complex translocated from the ER to the Golgi apparatus, which is required for optimal NLRP3 inflammasome assembly and activation [197]. In addition, NLRP3 promotes the expression of SREBP-1 and downstream proteins, as siRNA silencing of NLRP3 decreases the SERBP-1 level [61]. This finding clearly indicates that acute cholesterol depletion in ER by statins decreases IL-1*β* secretion, abrogates caspase-1 activation, and ablates NLRP3 inflammasome assembly, further solidifying the fact that ER sterol synthesis and distribution are principal determining factors for the activation of the NLRP3 inflammasome [189]. Cholesterol-dependent cytolysins induce mature IL-1 release from macrophages rapidly in a NLRP3 inflammasome- and cathepsin B-dependent manner [198].

Cholesterol crystals can activate the NLRP3 inflammasome and increase the production of IL-1*β* in monocytes/macrophages, as well as employing the complement system to induce cytokines and activate the inflammasome/caspase-1. It is noteworthy that the interaction between cholesterol crystals and the NLRP3 inflammasome is closely associated with AS [62, 79, 81, 199–205]. The involvement of the SREBP pathway in lipid synthesis plays a noticeable role in coordinating the relationship between the NLRP3 inflammasome-induced inflammatory response, lipid metabolism, and AS. Results from *in vitro* and *in vivo* studies suggest that SREBP-2 can aggravate endothelial dysfunction which is an important factor in AS [60]. Several studies reported that atheroprone flow induces marked proinflammatory response and oxidative stress in endothelial cells mediated through the SREBP-2-elicited NLRP3 inflammasome [62, 206]. This innate immune enhancement of the endothelium synergizes with hyperlipidemia, which leads to the topographic distribution of atherosclerotic lesions [62]. In conclusion, the SREBP-induced NLRP3 inflammasome and the innate immunity it stimulates are important contributors to AS and targeting SREBP-inflammasome pathways may be a therapeutic strategy for AS treatment [60].

What we need to emphasize here is that the nuclear respiratory factor-1 (NRF-1) which is targeted to the ER membrane and the UPR sensor proteins may regulate similar cellular processes [207]. Through a defined domain, NRF-1 directly binds to and specifically senses cholesterol in the ER, defending against cholesterol accumulation [208]. Therefore, it is an appealing notion that SREBP-2 and NRF-1 may be involved in a yin-yang counterbalance to stabilize cholesterol homeostasis in the ER. In addition, NRF-1 is a major transcriptional regulator that plays an essential role in integrating the transcription of nuclear-encoded mitochondrial genes involved in the mitochondrial respiratory chain and mitochondrial biogenesis [209–212]. Since NRF-1 plays an antioxidative role and the UPR is closely related to oxidative stress, it seems worthwhile to explore both roles in balancing the oxidative stress response [213, 214]. Several studies have indicated that proteasome disruption leads to ER stress and NRF-1 may mediate the proteasome recovery pathway after proteasome inhibition [215, 216]. The proteasome, UPR, and ERAD are transcriptionally integrated into the ER homeostasis pathway. NRF-1 regulates protein homeostasis in the ER through transcriptional regulation of ATF6, which regulates ERAD-associated gene expression, reducing the flow of protein substrates to the proteasome [207]. A NRF1-dependent increase in proteasome levels serves to influence the rate of new protein synthesis due to the increase in the intracellular pool of amino acids [217]. Together, NRF-1 can promote cholesterol removal and proteasome recovery, as well as antioxidant stress, all of which are beneficial to reducing ER stress. In other words, NRF-1 can counteract the adverse effects of the UPR. Although ER stress activates NRF-1, its specific mechanism has not been clarified [207, 214]. Given the possible beneficial effects of NRF-1 on ER stress and the subsequent inflammatory response, we need to further explore its possible role in alleviating inflammasome activation.

## 5. Conclusion and Perspectives

The ER maintains cellular functions through multiple pathways. Likewise, ER can produce a variety of adverse effects under stress. Although the UPR has long been recognized as a major effector mechanism of ER stress, it cannot be ignored that the ER, as an important site of intracellular calcium storage and lipid synthesis, is essential for maintaining calcium and lipid homeostasis. Therefore, ER stress inevitably disturbs calcium and lipid metabolism through downstream signaling pathways, resulting in a series of adverse effects. However, the mechanistic effects are not fully appreciated.

There have been many studies that have elucidated the mechanism of NLRP3 inflammasome activation. However, we need to be clear that the stimulatory signals required for NLRP3 inflammasome activation act at different stages of activation and the effects they produce are diverse. Although ER stress can activate the inflammasome, it is noteworthy that Menu et al. demonstrated that this effect is not directly affected by IRE1, PERK, ATF6, and its downstream TRAF2 and ASK1 in the classical UPR pathway but by other mechanisms [218]. As we reviewed above, ER stress has multiple effects on the activation of the NLRP3 inflammasome. Namely, it can directly affect the expression of terminal signaling in the UPR or stimulate activation through calcium or lipid metabolism or take effect through the production of ROS. ER stress seems to be underestimated in the significance of its contribution toward NLRP3 inflammasome activation. By summarizing the multiple mechanisms of ER stress-induced activation of the inflammasome, we clarify the important potential of this organelle in regulating the inflammasome-induced inflammatory response, which lays a foundation for further investigations. Finally, we conclude that the cross talk between ER stress and the NLRP3 inflammasome is related to AS. This review offers a fresh perspective where not only ER stress and the NLRP3 inflammasome but also the signaling hubs between them are potential intervention targets against AS worthy of further research.
